# Concordance and Clinical Significance of Genomic Alterations in Progressive Tumor Tissue and Matched Circulating Tumor DNA in Aggressive-variant Prostate Cancer

**DOI:** 10.1158/2767-9764.CRC-23-0175

**Published:** 2023-11-03

**Authors:** Ruiliang Wang, Qiufan Xu, Hanxu Guo, Guanjie Yang, Jun Zhang, Hong Wang, Tianyuan Xu, Changcheng Guo, Jing Yuan, Yanyan He, Xiaoying Zhang, Hongliang Fu, Guang Xu, Binghui Zhao, Jun Xie, Tingting Zhao, Longfei Huang, Jiansheng Zhang, Bo Peng, Xudong Yao, Bin Yang

**Affiliations:** 1 Department of Urology, Shanghai Tenth People's Hospital, Tongji University School of Medicine, Shanghai, P.R. China.; 2 Urologic Cancer Institute, Tongji University School of Medicine, Shanghai, P.R. China.; 3 Department of Urology, Shanghai Fifth People's Hospital, Fudan University, Shanghai, P.R. China.; 4 Department of Pathology, Shanghai Tenth People's Hospital, Tongji University School of Medicine, Shanghai, P.R. China.; 5 Department of Nuclear Medicine, Shanghai Tenth People's Hospital, Tongji University School of Medicine, Shanghai, P.R. China.; 6 Department of Nuclear Medicine, Xin Hua Hospital, Shanghai Jiao Tong University School of Medicine, Shanghai, P.R. China.; 7 Department of Medical Ultrasound, Center of Minimally Invasive Treatment for Tumor, Shanghai Tenth People's Hospital, Tongji University School of Medicine, Shanghai, P.R. China.; 8 Department of Radiology, Shanghai Tenth People's Hospital, Tongji University School of Medicine, Shanghai, P.R. China.; 9 Department of Urology, Shanghai Clinical College, Anhui Medical University, Shanghai, P.R. China.; 10 Research Institute, GloriousMed Clinical Laboratory, Shanghai, P.R. China.; 11 Department of Urology, Shanghai Tenth People's Hospital, Nanjing Medical University, Nanjing, P.R. China.

## Abstract

Sequencing of circulating tumor DNA (ctDNA) is a minimally invasive approach to reveal the genomic alterations of cancer; however, its comparison with sequencing of tumor tissue has not been well documented in real-world patients with aggressive-variant prostate cancer (AVPC). Concordance of genomic alterations was assessed between progressive tumor tissue and matched ctDNA by next-generation sequencing for 63 patients with AVPC. Associations of genomic alterations with progression-free survival (PFS) and overall survival (OS) were investigated using Kaplan–Meier and Cox regression analyses. A total of 161 somatic mutations (SMs) and 84 copy-number variants (CNVs) were detected in tumors, of which 97 were also found in ctDNA, giving concordance of 39.6% (97/245) across all SMs and CNVs, 49.7% for SMs only and 20.2% for CNVs only. Across all patients with AVPC, chemotherapy was associated with significantly longer median PFS (6 vs. 0.75 months, *P* = 0.001) and OS (11 vs. 8 months, *P* < 0.001) than next-generation hormonal therapy (NHT). Among types of chemotherapy, additional platinum-based chemotherapy was associated with significantly longer median PFS and OS than docetaxel only in patients with *TP53*, *RB1*, or *PTEN* alterations, and in those with ctDNA% ≥ 13.5%. The concordance analysis first provides evidence for combining the sequencing of ctDNA and tumor tissue in real-world patients with AVPC. Chemotherapy is associated with significantly better survival than NHT, and the benefit of additional platinum-based chemotherapy may depend on the presence of alterations in *TP53*, *RB1*, or *PTEN* and on a sufficiently high proportion of ctDNA in patients with AVPC.

**Significance:**

AVPC is a highly malignant and heterogeneous disease. Sequencing of ctDNA is a minimally invasive approach to reveal genomic alterations. On the basis of the current real-world study, we found ctDNA does not fully recapitulate the landscape of genomic alterations from progressive tumor tissue in AVPC. We also revealed AVPC can benefit from chemotherapy, especially platinum-based regimens. *TP53/RB1/PTEN* alterations in ctDNA or tumor tissue could be biomarkers for platinum-based chemotherapy in this setting.

## Introduction

Aggressive-variant prostate cancer (AVPC) is characterized by androgen independence, relatively low levels of PSA, and aggressive clinical course involving early and extensive visceral metastasis, transient response to conventional androgen deprivation treatment (ADT) and poor survival (1). The prevalence of AVPC among patients with castration-resistant prostate cancer (CRPC) has been on the rise and may be as high as 20%, which likely reflects selective pressure due to the increasing use of next-generation hormonal therapy (NHT; refs. 2–4). AVPC usually responds initially to combinational platinum-based chemotherapy but not over a longer period, and most treated patients die within 12--24 months after diagnosis (5–7). Thus, we need to understand more about how genomic alterations in AVPC influence treatment response and prognosis (5, 8, 9).

Next-generation sequencing (NGS) of tumor tissue can reveal the genomic alterations of cancer to personalize treatment and predict survival (6, 9–14). For example, alterations in at least two of the tumor suppressor genes *TP53*, *RB1*, and *PTEN* have been used to classify AVPC into treatment groups (15). However, analyzing tumor tissue has major disadvantages. One is the spatial heterogeneity, which means that tumor biopsy or surgery may not reflect the cellular composition elsewhere in the patient (16, 17). Another is that formalin-fixed, paraffin-embedded tumor tissue that has been stored for longer periods may not give useful sequencing results (11). A third disadvantage, as for the degree of malignancy of tumor, is that aggressive lesions always have more alterative frequency than regressive lesions. But molecular changes detected using one tissue samples reflect only a snapshot along the cancer progression and development of treatment resistance, and conventional imaging, such as CT and bone scintigraphy, has difficulties in recognizing the progressive and lethal lesions in patients with AVPC (2, 18–21).

An alternative source of tumor DNA for characterizing its genomic alterations may be the blood, in the form of circulating tumor DNA (ctDNA; refs. 2, 16, 22). This analysis does not require tumor biopsy or surgery and it detects DNA from primary and metastatic tumors. The reliability of ctDNA sequencing as a tool for predicting prognosis and treatment response depends on the concordance between the alterations detected in ctDNA and those detected in tumor tissue. For high tumor burden CRPC, this concordance is known to be high (16, 22–25). Whether the same is true for AVPC is unclear. In addition, detecting copy-number variants (CNV), which often occur in AVPC, can be difficult from ctDNA samples if a low proportion of the circulating DNA actually comes from tumors (16, 24, 26).

Previous work has suggested that analyzing ctDNA from patients with AVPC can predict response to carboplatin chemotherapy (6). To validate the use of such sequencing for personalizing treatment and predicting prognosis of patients with AVPC, we assessed here the concordance of genomic alterations between progressive tumor tissue and matched ctDNA. Our findings from real-world data suggest that ctDNA sequencing on its own may not be sufficiently reliable or informative for clinical decision-making. We also explored the association of genomic alterations with patient survival to select patients with AVPC who can benefit from chemotherapy, especially platinum-based chemotherapy.

## Materials and Methods

### AVPC Clinical Criteria

Diagnosis of AVPC was determined by one or more of the clinical criteria which were first introduced by Aparicio (7): (i) any histologic evidence of small cell carcinoma; (ii) exclusively visceral metastases; (iii) radiographically predominant lytic bone metastases diagnosed by CT; (iv) bulky lymphadenopathy or bulky (≥5 cm, respectively) high-grade (Gleason score≥ 8) tumor mass in prostate/pelvis; (v) low PSA level (≤10 ng/mL) at initial presentation (prior to ADT or at symptomatic progression in the castrate status) and high volume (≥20) of bone metastases; (vi) presence of neuroendocrine markers on histology (chromogranin A and synaptophysin) combined with either elevated serum lactate dehydrogenase (LDH) value, malignant hypercalcemia, or elevated serum carcinoembryonic antigen value (all in absence of other causes); (vii) short interval (≤6 months) to androgen-independent progression following the initiation of ADT. The presence of clinical criteria for AVPC was shown in Supplementary Fig. S1.

### Patients and Samples

This study retrospectively analyzed data for 63 consecutive patients with AVPC who were treated at Shanghai Tenth People's Hospital (Shanghai, P.R. China) between January 2018 and August 2021. The progressive tumor tissues were obtained through biopsy or surgery, and ctDNA from the peripheral blood was collected within 1 week before biopsy or surgery. The progressive lesions were identified by a multidisciplinary team of physicians from Urology, Radiology, Nuclear Medicine, Ultrasound Medicine and Pathology based on multimodal imaging, and the oligoprogression was defined as five or less than five progressive lesions. Polyprogression was defined as more than five progressive lesions (27).

**FIGURE 1:**
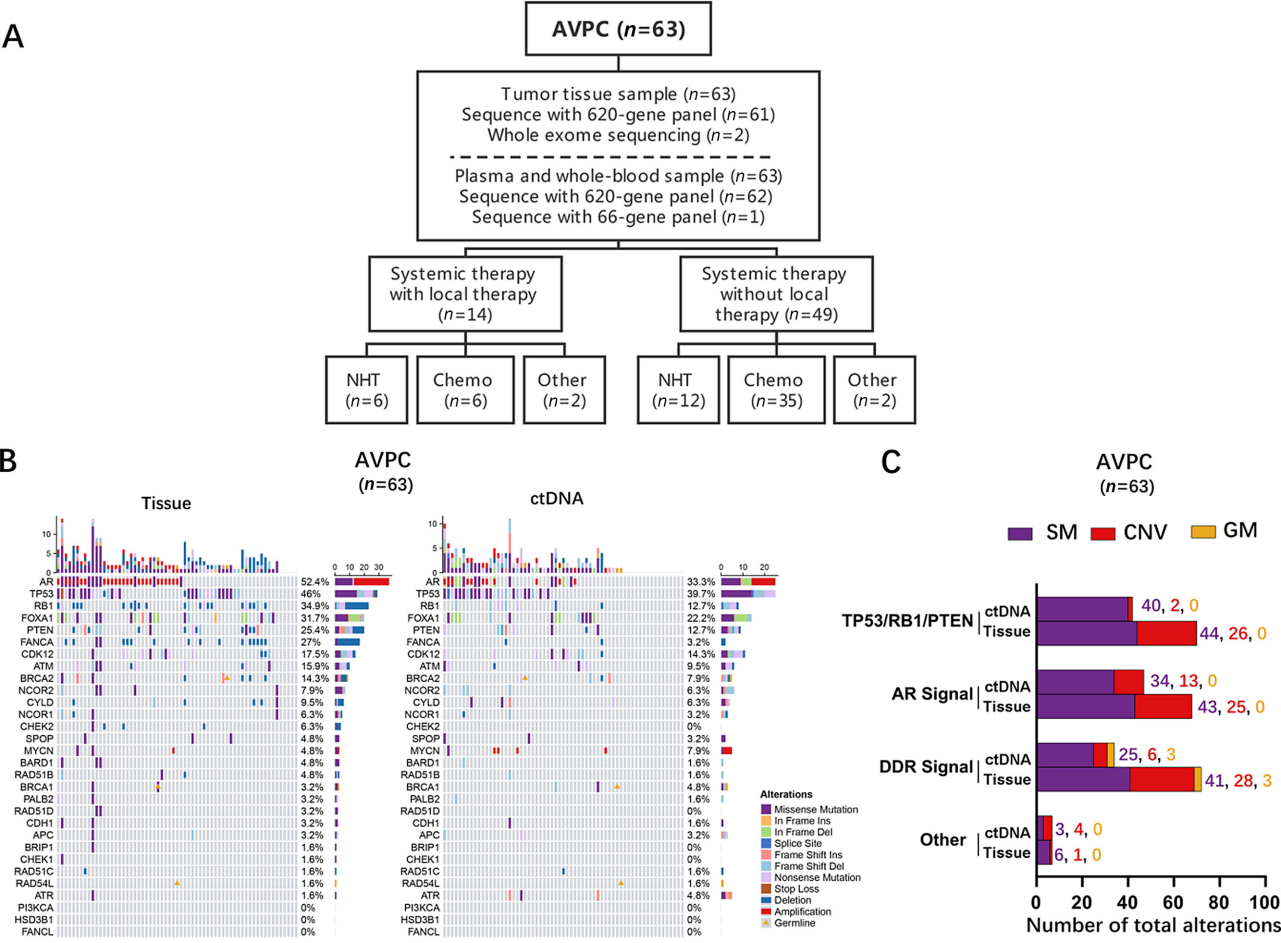
Study design and integrative landscape of SMs, CNVs, and deleterious GMs in patients with AVPC. **A,** Flow diagram of patients according to treatment history. Tumor tissue and matched ctDNA from 63 patients with clinically defined AVPC. All of them underwent systemic treatment. Among them, 14 patients received local treatment, while 59 patients did not. The systemic treatment methods included NHT (enzalutamide, abiraterone, and apalutamine), Chemotherapy (additional platinum-based chemotherapy or docetaxel only), and other therapies (inhibitors of tyrosine kinases, PD-1 or PARP inhibitor). **B,** Landscape of alterations involving 30 selected genes in progressive tumor tissues and matched ctDNA from patients with AVPC. **C,** Landscape of alterations involving signaling pathways in progressive tumor tissues and matched ctDNA from patients with AVPC. Significance was determined using *χ*^2^ and Fisher exact tests. ***, *P* < 0.001.

Patient sampling and treatment are shown in Fig. 1A and Supplementary Fig. S1. NHT in the present study involved enzalutamide, abiraterone, and apalutamine. Additional platinum-based chemotherapy was used in combination with docetaxel in patients with no prior docetaxel treatment, or with etoposide in patients after docetaxel treatment. Platinum-based chemotherapy was also used alone in some selected patients who had disease progressed after docetaxel. Platinum-based chemotherapy includes cisplatin and carboplatin.

This study received approval from the Ethics Committee of Shanghai Tenth People's Hospital (SHSY-IEC-5.0/22K50/P01). The investigators obtained written informed consent from the subjects in accordance with the Declaration of Helsinki for the analysis and publication of their anonymized medical data for research purposes at the time of treatment.

### DNA Sequencing and Bioinformatics

The targeted NGS using progressive tumor tissue and matched ctDNA was performed at GloriousMed Clinical Laboratory according to the standard protocol as described in our previously published articles (28, 29). Briefly, we used whole-exome sequencing or targeted sequencing strategy capturing the exons of 620 or 66 prostate cancer driver genes in progressive tumor tissue and ctDNA samples. For each sample, 20 to 100 ng of cell-free DNA, 200 to 500 ng of formalin-fixed, paraffin-embedded tumor DNA, or 500 ng of genomic DNA was used for library preparation and quantification. Trimmomati (30) was used to trim the sequencing adapters from the raw data. All of the individuals were sequenced at a depth of at least 10X (average sequencing depth = 22X). The reads were aligned with the human reference genome (hg19) using Burrows-Wheeler Aligner (31). Duplicated reads were removed using Picard (http://broadinstitute.github.io/picard/). Mapped reads were realigned to the genome using Genome Analysis Tool Kit (32). Somatic mutations (SM) and germline mutations (GM) were called using Mutect2 and GATK's Haplotype Caller (32) with a paired workflow, respectively. Variants were then annotated using ANNOVAR (33) and in-house developed code. An in-house script was used to verify the human identity concordance of paired samples. Somatic copy-number alterations were also detected using GATK (32). We focused on the 30 genes for comprehensive study (*TP53*, *RB1*, *PTEN*, *six* genes in AR signal pathway, 17 genes in DNA-damage repair (DDR) signal pathway, *PI3KCA*, *MYCN*, *CYLD*, and *APC*). Bioinformatics analysis of the sequencing data, including identification of GMs, SMs, and CNV, was performed according to the published standards (34, 35).

### Concordance in Genomic Alterations Between ctDNA and Progressive Tumor Tissue

Concordance analysis was based on 30 most altered genes in progressive tumor tissues associated with prostate cancer. The concordance was estimated on the basis of SM plus CNV, SM, and CNV between progressive tumor tissue and matched ctDNA, respectively as follows:

First, the overall concordance rate was calculated by the sum of all concordant alterant sites between progressive tumor tissue and matched ctDNA dividing the sum of alterant sites detected in progressive tumor tissue. Second, the positive-concordance score was used for evaluating the individual concordance which was calculated as the number of concordant genes between progressive tumor tissue and matched ctDNA in a patient divided by the number of genes detected in the progressive tumor tissue. If the positive-concordance score ≥50%, it was identified as high positive-concordance score or else identified as low positive-concordance score. The association between individual positive-concordance score and clinical characteristics was investigated and the clinical characteristics affecting the positive-concordance score were further analyzed. Negative concordance was defined as no detected alterations in neither progressive tumor tissue nor matched ctDNA. Third, the concordance analysis was also performed for *TP53/RB1/PTEN* alterations in patients with AVPC, and the percentage of positive concordance (PPC) and percentage of negative concordance (PNC) were further calculated according to the methods reported previously (36). The individual positive concordance of *TP53/RB1/PTEN* was defined as presence of any *TP53/RB1/PTEN* alteration in both progressive tumor tissue and matched ctDNA. The individual negative concordance of *TP53/RB1/PTEN* was defined as absence of any *TP53/RB1/PTEN* alteration in neither progressive tumor tissue nor matched ctDNA. PPC was calculated by the number of patients with positive concordance of *TP53/RB1/PTEN* alterations between progressive tumor tissue and matched ctDNA dividing the number of patients with *TP53/RB1/PTEN* alterations in progressive tumor tissue. PNC was calculated by the number of patients with negative concordance of *TP53/RB1/PTEN* alterations in progressive tumor tissue and matched ctDNA dividing the number of patients without *TP53/RB1/PTEN* alteration in progressive tumor tissue.

All the pathogenic variants, likely pathogenic variants, benign variants, likely benign variants, and variants of unknown significance were used for concordance calculation. The pathogenic variants and likely pathogenic variants were used for the survival analysis.

### Survival Outcomes

Patients were closely followed up every 3–4 weeks after therapy started. At follow-up visits, the tumor biomarkers, such as PSA, were detected, and the progressive lesions were assessed using multimodal imaging. Disease stage and regression or progression were assessed on the basis of Prostate Cancer Working Group 3.0 criteria and RECIST 1.1 criteria. Patients were followed up until death or September 31, 2021. Progression-free survival (PFS) was defined as the time from treatment initiation to confirmed biochemical recurrence, clinical or radiographic progression or death (37, 38). Overall survival (OS) was defined as the time between treatment initiation and death from any cause. PSA response was defined as a decline from baseline level by at least 50% at any time during follow-up (37, 38).

### Statistical Analysis

Data were analyzed using Graphpad Prism 8 (RRID:SCR_002798), SPSS 25.0 (RRID:SCR_016479), or R 3.6.1 (RRID:SCR_000432). Results associated with two-sided *P* < 0.05 were considered statistically significant unless otherwise noted. Different frequencies of genomic alterations between patients who received local therapy (surgery and/or radiotherapy) or not were assessed for significance using Student *t* test. PFS and OS were analyzed using the Kaplan–Meier method, and curves were compared using the log-rank test. *P* values by multiple testing were adjusted using Benjamini and Hochberg (39). Risk factors for poor survival were identified by conducting univariate analysis, the significant factors from which (based on *P* < 0.01) were entered into multivariate Cox proportional hazards regression. Results were reported as HRs and 95% confidence intervals (CI).

### Data Availability

The data generated in this study are not publicly available because of patient privacy but are available upon reasonable request in accordance with Chinese regulations on management of human genetic resources policy. However, interested parties can obtain this data by making a reasonable request to the corresponding author. The remaining data generated in this study are accessible in the article and its Supplementary Data.

## Results

### Patient Characteristics

There were 63 patients with AVPC included in this study (Fig. 1A), and their clinical characteristics were summarized in Table 1 and Supplementary Fig. S1. The clinical characteristics and imaging results used to verify progressive lesions for each patient were shown in Supplementary Table S1. There were 37 patients (37, 58.7%) with a Gleason score > 8 at initial diagnosis. Median time from initial diagnosis to CRPC was 9.0 months (interquartile range, 5.0 to 25.0 months). The median proportion of total DNA in circulation that came from tumors (hereafter referred to as ctDNA%) was 15% (interquartile range, 5 to 38.0).

**TABLE 1:** Clinical Characteristics of Patients with AVPC

Characteristic	AVPC (*n* = 63)
Initial diagnosis	
PSA	
Median (IQR)	66.17 (24.52–161.86)
Gleason score, *n* (%)	
≤8	22 (34.92%)
>8	37 (58.73%)
Unknown	4 (6.35%)
T stage, *n* (%)	
≤T3a	2 (3.17%)
>T3a	61 (96.83%)
N stage, *n* (%)	
N0	4 (6.34%)
N1	59 (93.65%)
M stage, *n* (%)	
M0	5 (7.94%)
M1	58 (92.06%)
Timepoint of NGS	
Age	
Mean ± SD	65.84 ± 8.89
Time to CRPC	
Median (IQR)	9 (5–25)
PSA	
Median (IQR)	7.8 (1.02–45.33)
Multimodality imaging, *n* (%)	
FDG-PET/CT	58 (92.06%)
PSMA-PET/CT	42 (66.67%)
CEUS of liver metastases	9 (14.29%)
mpMRI	61 (96.83%)
Bone scintigraphy or CT	7 (11.11%)
Metastasis status, *n* (%)	
Oligoprogressive lesion	21 (33.33%)
Polyprogressive lesion	42 (66.67%)
Site of progressive lesion, *n* (%)	
Bone	8 (12.70%)
Viscera (Lung, Liver, Adrenal gland)	11 (17.46%)
Lymph node	8 (12.70%)
Prostate gland or bed	28 (44.44%)
Bladder neck	8 (12.70%)
ECOG, *n* (%)	
0	45 (71.44%)
1	16 (25.40%)
2	2 (3.18%)
ctDNA%	
Median (IQR)	0.15 (0.05–0.38)
Received treatment line for CRPC at time of ctDNA and tissue collection, *n* (%)	
Treatment-naïve	24 (38.10%)
First-line treatment	27 (42.86%)
Second-line or later-line treatment	12 (19.05%)
Median follow-up time from initial diagnosis	
Median (IQR)	33 (15.5–52)

Abbreviations: AVPC, aggressive-variant prostate cancer; CEUS, contrast-enhanced ultrasonographic imaging of liver metastases; CRPC, castration-resistant prostate cancer; CT, computerized tomography; IQR, interquartile range; mpMRI, multiparametric magnetic resonance imaging; NGS, next-generation sequencing; PSA, prostate-specific antigen; PSMA, prostate-specific membrane antigen.

### Landscape of Genomic Alterations in Tumors and ctDNA

In progressive tumor tissues, the genes most frequently affected by GMs, SMs, or CNVs were androgen receptor (*AR*) (52.4% of patients), *TP53* (46.0%), *RB1* (34.9%), *FOXA1* (31.7%), and *PTEN* (25.4%; Fig. 1B). In ctDNA, the genes most frequently affected were *TP53* (39.7%), *AR* (33.3%), *FOXA1* (22.2%), *CDK12* (14.3%), *PTEN* (12.7%), and *RB1* (12.7%). The proportions of patients whose progressive tumor tissue showed alterations (especially CNVs) in the genes *TP53*, *RB1*, and *PTEN* or in the genes involved in AR signaling pathways or in DDR were considerably higher than the corresponding proportions of patients whose ctDNA showed alterations in these genes (Fig. 1C).

### Concordance of Alterations Between Tumors and ctDNA

**FIGURE 2:**
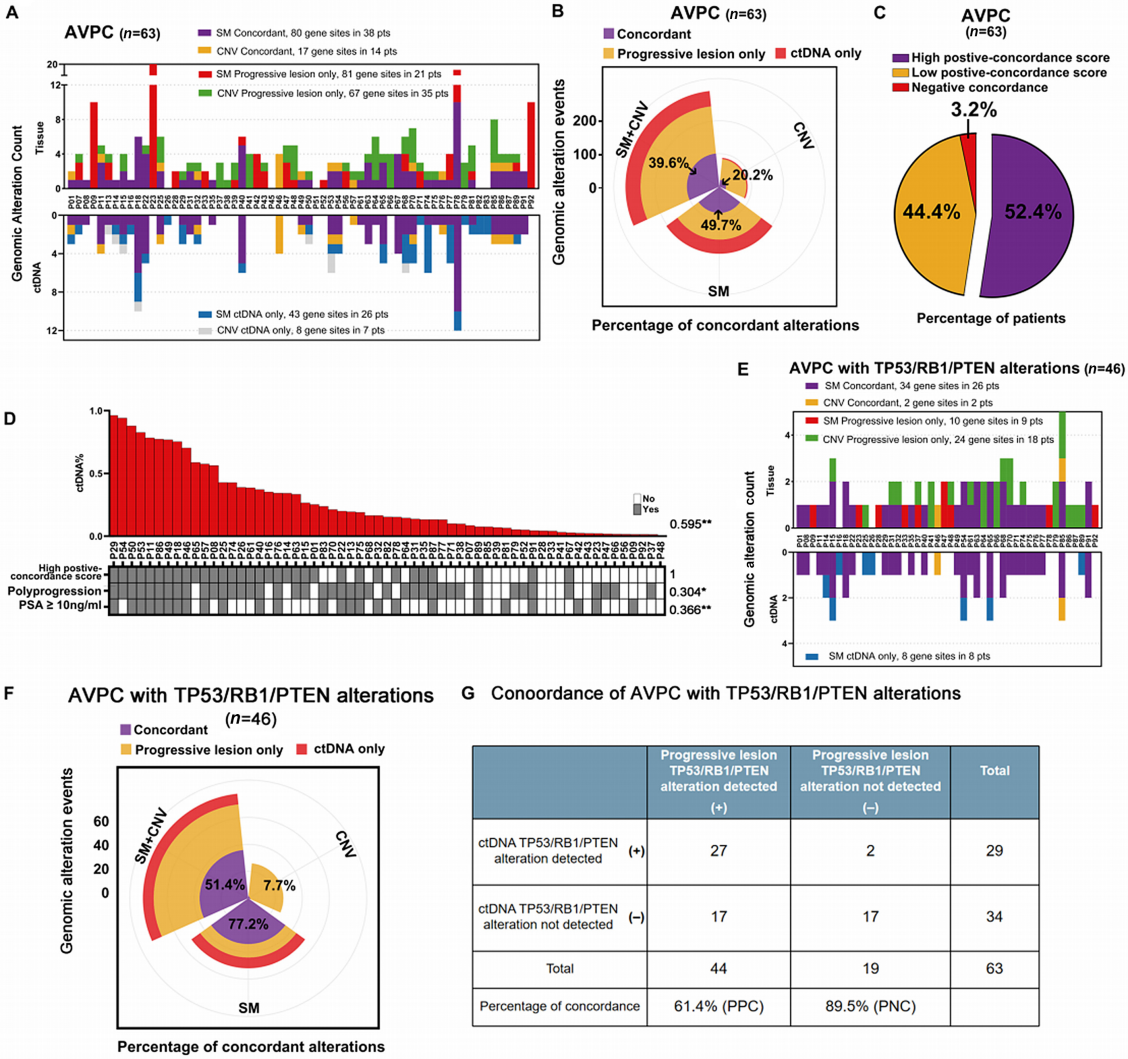
Concordance of genomic alterations between proggressive tumor tissue and matched ctDNA from patients with AVPC. **A,** Concordance in SMs and CNVs. Data are shown for 63 patients. **B,** Rose diagram of concordance for SMs + CNVs, SMs only or CNVs only. **C,** Proportions of patients with high positive concordance, low positive concordance or negative concordance. **D,** Association between high positive concordance and clinical characteristics, including the ctDNA%, AVPC criteria, progressive status, and PSA ≥ 10 ng/mL at the time of DNA sequencing. Associations were assessed using Kendall tau test, and significance was defined as *, *P* < 0.05 or **, *P* < 0.01. Data are shown for 61 patients. **E–G,** Concordance of alterations involving the tumor suppressor genes *TP53*, *RB1*, or *PTEN* in 46 patients with AVPC. **E,** Concordance of SMs or CNVs. **F,** Rose diagram of concordance for SMs + CNVs, SMs only or CNVs only. **G,** Proportions of patients with positive or negative concordance. ALP, alkaline phosphatase.

The complete list of genomic alterations detected in progressive tumor tissues and ctDNA for each patient was shown in Supplementary Tables S2 and S3. Altogether 80 SMs (in 38 patients) and 17 CNVs (in 14 patients) were concordant, 81 SMs in 21 patients and 67 CNVs in 35 patients were found only in tumor tissues, and 43 SMs in 26 patients and 8 CNVs in 7 patients were found only in ctDNA (Fig. 2A). Across all 63 patients, concordance was 39.6% for the combination of SMs and CNVs (97/245), 49.7% for only SMs (80/161) and 20.2% for only CNVs (17/84; Fig. 2B). When we considering the patients who have alteration detections in both tumor tissue and ctDNA in terms of the same alteration type, the concordance is 58.8% in SM+SNV, 77.7% in SM and 42.5% CNV (Supplementary Fig. S2).

At the level of individual patients, concordance was ≥50% in 52.5% of patients (33/63), while it was <50% in 44.4% of patients (28/63; Fig. 2C). After excluding 2 patients who showed negative concordance (no alteration in either tumor tissue or ctDNA), univariate regression analysis of the remaining 61 patients showed that having concordance≥ 50% correlated positively with higher ctDNA% (Kendall tau-b = 0.595, *P* < 0.01), polyprogression (Kendall tau-b = 0.340, *P* = 0.013), and PSA ≥ 10 ng/mL (Kendall tau-b = 0.366, *P* < 0.01; Fig. 2D). Multivariate logistic regression linked having a high concordance score with higher ctDNA% (HR, 1.173; 95% CI, 1.084–1.284; Table 2). We found that 13.5% was the optimal cutoff at which ctDNA% predicted concordance ≥ 50%, which gave a sensitivity of 84.8% and specificity of 78.6% (Supplementary Fig. S3).

**TABLE 2:** Univariate and multivariate logistic regression to identify associations between high positive concordance and clinical characteristics

		AVPC			
		Univariate analysis		Multivariate analysis	
Characteristics	Total (*N*)	OR (95% CI)	*P* value	OR (95% CI)	*P* value
Metastasis status			0.021		0.638
Oligometastasis	19	Reference		Reference	
Polymetastasis	42	3.90 (1.229–12.379)		1.513 (0.270–8.484)	
PSA at NGS			0.006	—	0.072
≥ 10 ng/mL	25	Reference		Reference	
< 10 ng/mL	36	4.976 (1.597–15.504)		4.722 (0.873–25.543)	
ctDNA%	61	1.173 (1.074–1.284)	<0.001	1.173 (1.074–1.284)	<0.001

Abbreviations: AVPC, aggressive-variant prostate cancer; ctDNA, circulating tumor DNA; NGS, next generation sequencing; OR, odds ratio; PSA, prostate specific antigen.

Among alterations affecting the genes *TP53*, *RB1*, and *PTEN*, 34 SMs in 26 patients and two CNVs in 2 patients were detected in progressive tumor tissues and ctDNA, 10 SMs in 9 patients and 24 CNVs in 18 patients were detected only in tumor tissues, and 8 SMs in 8 patients were detected only in ctDNA. Across all 63 patients, concordance was 51.4% for the combination of SMs and CNVs (36/70), 77.2% for only SMs (34/44), and 7.7% (2/26) for only CNVs (Fig. 2E and F). The PPC was 61.4% and PNC was 89.5% (Fig. 2G; Supplementary Fig. S4).

### Genomic and Clinical Characteristics Associated with Treatment Response and Survival

**FIGURE 3:**
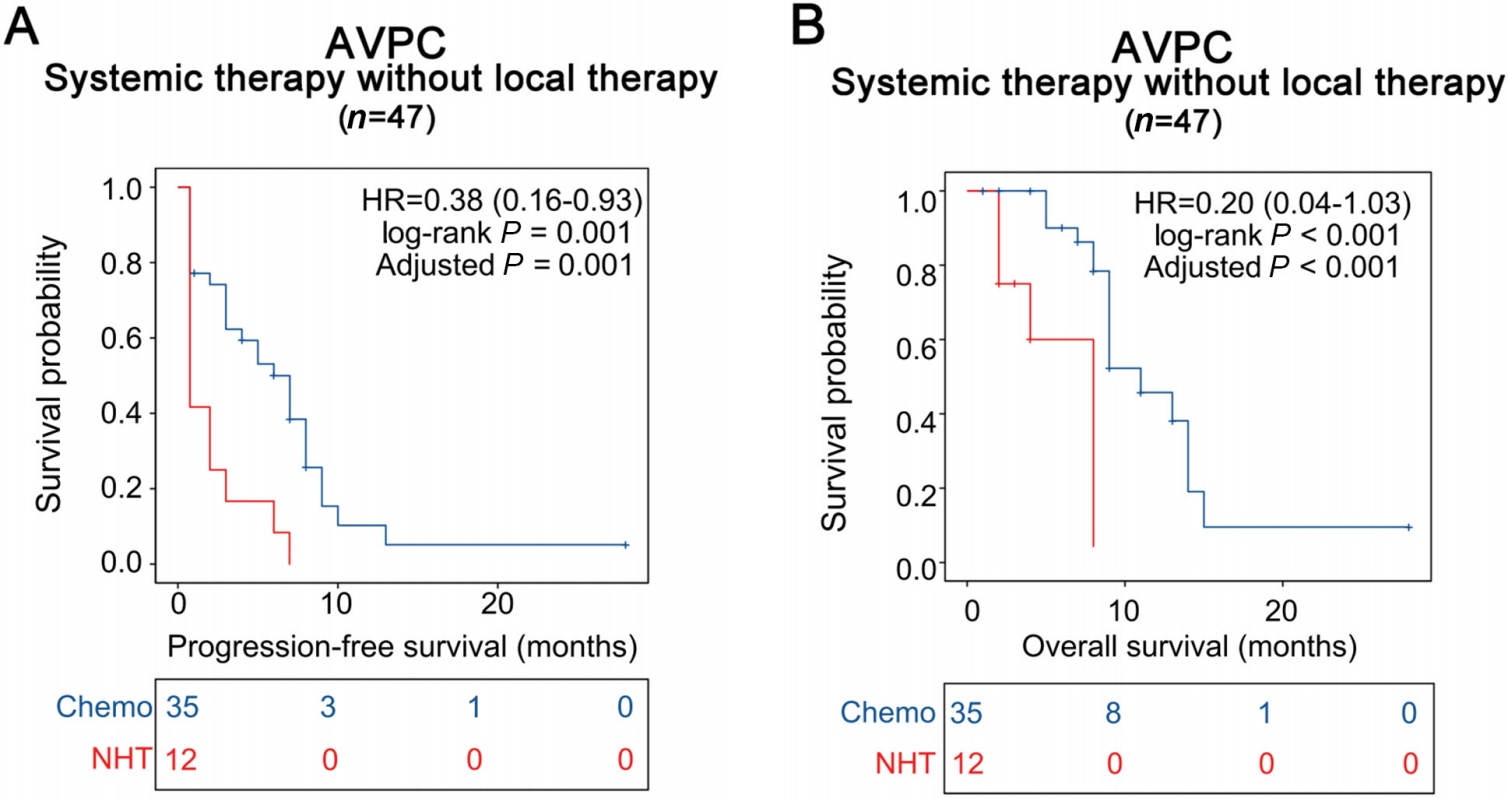
Patients with AVPC demonstrate improved survival outcomes during Chemo compared with NHT. PFS (**A**) and OS (**B**) of patients with AVPC stratified of NHT or Chemo.

The rate of PSA response was 55.8% (29/52), the rate of 6-month PFS was 49.2% (31/63), and the rate of 12-month OS was 22.2% (14/63; Fig. 1A; Supplementary Table S4). Compared with patients treated with NHT, those treated with chemotherapy had significantly longer median PFS (6 vs. 0.75 months; HR, 0.38; 95% CI, 0.16–0.93; *P*_adjusted_ = 0.001) and OS (11 vs. 8 months; HR, 0.20; 95% CI, 0.04–1.03; *P*_adjusted_ < 0.001; Fig. 3A and B). Univariate Cox regression involving 49 patients who received systemic therapy without local therapy associated the presence of alterations affecting *TP53*, *RB1*, or *PTEN* with shorter PFS and OS (Supplementary Figs. S5 and S6). Multivariate regression of the same 49 patients, which included alterations affecting *TP53*, *RB1*, or *PTEN*, metastasis status, visceral metastasis, PSA at NGS, Eastern Cooperative Oncology Group (ECOG), line of treatment after NGS, alkaline phosphatase, LDH, hemoglobin, ctDNA% and numbers of AVPC diagnostic components (7) fulfilled by the patient, identified two variables as independent predictors of PFS and OS (Table 3): alterations affecting *TP53, RB1* or *PTEN;* and ctDNA%.

**TABLE 3:** Univariate and multivariate Cox analysis of PFS and OS in patients with AVPC who received systemic therapy without local therapy

		PFS Univariate analysis		PFS Multivariate analysis		OS Univariate analysis		OS Multivariate analysis	
Characteristics	Total (*N*)	HR (95% CI)	*P* value	HR (95% CI)	*P* value	HR (95% CI)	*P* value	HR (95% CI)	*P* value
*TP53/RB1/PTEN*	49		0.041				0.011		
No alteration	13	Reference		Reference		Reference		Reference	
Any alteration	36	2.209 (1.032–4.729)		2.483 (1.095–5.629)	0.029	3.134 (1.140–8.615)		4.121 (1.317–12.900)	0.015
Metastasis status	49		0.078				0.327		
Oligometastasis	7	Reference		Reference		Reference			
Polymetastasis	42	1.846 (0.768–4.439)		1.804 (0.681–4.781)	0.235	1.726 (0.580–5.136)			
Visceral metastasis							0.462		
No	18					Reference			
Yes	31					1.395 (0.580–3.357)			
PSA at NGS	49		0.653				0.836		
≤10 ng/mL	27	Reference				Reference			
>10 ng/mL	22	1.154 (0.618–2.154)				1.092 (0.474–2.514)			
ECOG	49		0.214				0.066		0.707
0	29	Reference				Reference		Reference	
1 & 2	20	1.484 (0.796–2.767)				2.176 (0.938–5.046)		1.188 (0.485–2.907)	
Line of treatment after NGS	49		0.513				0.565		
Treatment-naïve	16	Reference				Reference			
First-line treatment	22	1.524 (0.743–3.122)	0.250			0.861 (0.325–2.284)	0.764		
Second- and later-line treatment	11	1.354 (0.568–3.226)	0.494			1.516 (0.502–4.580)	0.461		
ALP	49	1.001 (0.999–1.002)	0.349			1.000 (0.997–1.002)	0.730		
LDH	49	1.000 (1.000–1.001)	0.320			1.001 (1.000–1.002)	0.150		
Hemoglobin	48	1.001 (0.987–1.015)	0.882			1.000 (0.983–1.017)	0.981		
ctDNA%	49	1.010 (1.000–1.021)	0.053	1.012 (1.000–1.024)	0.049	1.019 (1.006–1.033)	0.005	1.023 (1.007–1.039)	0.004
Quantity of AVPC criteria meet	49		0.978				0.622		
1	22	Reference				Reference			
2	11	0.851 (0.386–1.875)	0.688			1.220 (0.415–3.580)	0.718		
3	10	0.848 (0.376–1.913)	0.692			1.457 (0.452–4.697)	0.528		
4	4	0.930 (0.269–3.209)	0.908			2.026 (0.514–7.984)	0.313		
5	2	0.576 (0.076–4.367)	0.593			4.606 (0.529–40.066)	0.166		

Abbreviations: ALP, alkaline phosphatase; AVPC, aggressive-variant prostate cancer; ctDNA, circulating tumor DNA; HR, hazard ratio; LDH, lactate dehydrogenase; NGS, next generation sequencing; OS, overall survival; PFS, profession-free survival; PSA, prostate specific antigen.

### Comparative Efficacy of Different Chemotherapies for Patients with High ctDNA% and Alterations Involving *TP53*, *RB1*, or *PTEN*

A total of 35 patients received chemotherapy: 13 (37.1%) received docetaxel only and 22 (62.9%) received additional platinum-based chemotherapy. Among those receiving docetaxel only, the rate of PSA response was 25.0% (3/12). The rate of 6-month PFS was 23.1% (3/13), and the rate of 12-month OS was 23.1% (3/13; Supplementary Fig. S1). The corresponding rates in patients receiving additional platinum-based chemotherapy were 70.6% (12/17), 59.1% (13/22), and 13.6% (3/22).

**FIGURE 4:**
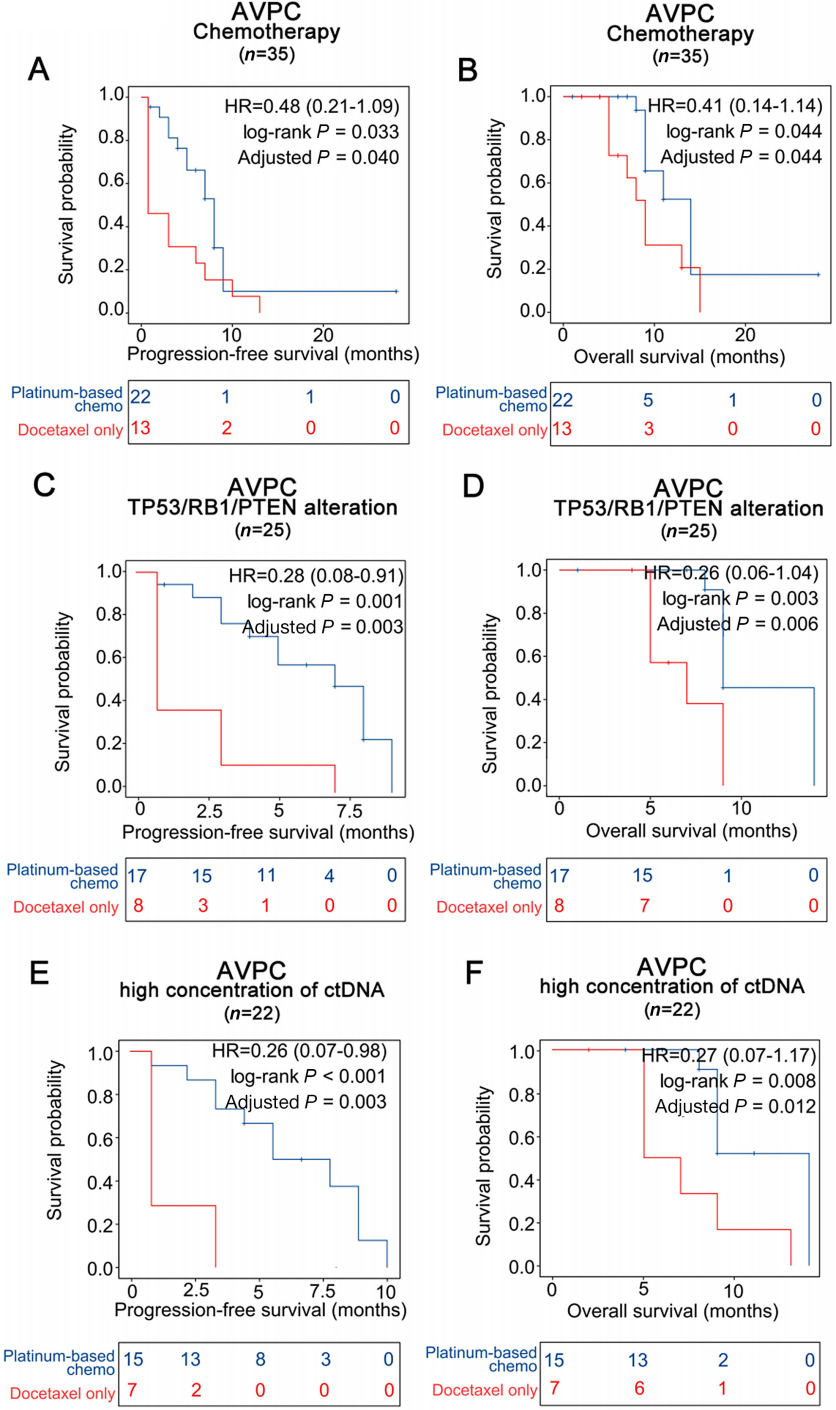
AVPC patient survival outcomes with additional platinum-based chemotherapy or docetaxel only based on *TP53/RB1/PTEN* alterations and high ctDNA%. PFS (**A**) and OS (**B**) of patients with AVPC who underwent additional platinum-based chemotherapy or docetaxel only, PFS (**C**) and OS (**D**) of patients with AVPC with *TP53/RB1/PTEN* alterations stratified of additional platinum-based chemotherapy or docetaxel only, and PFS (**E**) and OS (**F**) with high ctDNA% (≥13.5%) stratified of additional platinum-based chemotherapy or docetaxel only.

Compared with patients receiving docetaxel only, those receiving additional platinum-based chemotherapy had significantly longer median PFS (8 vs. 0.75 months; HR, 0.48; 95% CI, 0.21–1.09; *P*_adjusted_ = 0.033) and OS (14 vs. 9 months; HR, 0.41; 95% CI, 0.41–1.14; *P*_adjusted_ = 0.044; Fig. 4A and B). Specifically, additional platinum-based chemotherapy had significantly longer median PFS and OS than docetaxel only in the subgroups of patients with alterations involving *TP53*, *RB1*, or *PTEN* (PFS, 7 vs. 0.75 months; HR, 0.28; 95% CI, 0.08–0.91; *P*_adjusted_ = 0.003; Fig. 4C; OS, 9 vs. 7 months; HR, 0.26; 95% CI, 0.06–1.04; *P*_adjusted_ = 0.006; Fig. 4D) and those with ctDNA% ≥ 13.5% (PFS, 6 vs. 0.75 months; HR, 0.26; 95% CI; 0.07–0.98; *P*_adjusted_ = 0.003; Fig. 4E; OS, 14 vs. 6 months; HR, 0.27; 95% CI, 0.07–1.07; *P*_adjusted_ = 0.012; Fig. 4F). However, no statistical difference was found in the survival outcomes between additional platinum-based chemotherapy and docetaxel only in the AVPC subgroups without any alteration involving the three tumor suppressor genes (*TP53*, *RB1*, or *PTEN*), or those with ctDNA% < 13.5% (Supplementary Fig. S7).

## Discussion

To our knowledge, this is the first real-world analysis of the genomic alterations in matched progressive tumor tissue and ctDNA in patients with AVPC. Our study suggests that concordance between alterations detected in tumors and in ctDNA is only moderate and depends on ctDNA proportion in the blood. Our results argue that ctDNA sequencing on its own is insufficient for fully characterizing the genomic alterations of AVPC tumors. The combination of sequencing progressive tumor tissue based on multimodal imaging as well as sequencing of ctDNA could provide a more complete molecular characterization of AVPC and predict the patient's prognosis and treatment response.

We found concordance of 49.7% in SMs and 20.2% in CNVs. For SMs, this number was low which means some patients don't have enough ctDNA% but have detection in progressive tissue. But if we consider the patients who have alternative detections in both tumor tissue and ctDNA, this number will increase to 77.7% which is similar to the previous results which reported concordance > 80% (26, 28, 40). For CNVs, the concordance was only 20.2% or 42.5% which most likely reflects the relatively small numbers of CNVs detected. Previous work has already shown that reliably detecting CNVs in PTEN and RB1 requires ctDNA% greater than 30% (16, 24, 41), much higher than the median ctDNA% of 15% in our cohort. Apart from this, previous work has shown that ctDNA% from more than 50% of patients with AVPC is insufficient for CNV analysis, whereas circulating tumor cells (CTC) are much more reliable and robust DNA sources for such analysis (42, 43). Both SMs and CNVs results proved that although AVPC was considered to be an aggressive disease that has high tumor burden, it is still not appropriate to regard ctDNA as a perfect substitution of progressive tissue based on our real-world study, especially in CNV. Novel techniques should be developed to further improve the sensitivity of ctDNA detection in the future (44).

On the other hand, our analysis suggests that sequencing of progressive tumor tissue and ctDNA are both necessary for obtaining a complete understanding of the genomic alterations of AVPC. More genomic alterations in AR and DDR signaling pathways were detected in tumor tissues than in ctDNA, while ctDNA also revealed genomic alterations not detected in tumor tissues, likely because ctDNA can better detect subclonal heterogeneity among tumor cells than a single biopsy or surgery tissue from part of the tumor itself (2, 45–48).

Alterations in AR signaling genes were frequent among our patients with AVPC, which is consistent with the literature on genomic alterations in patients from other ethnic groups (Supplementary Fig. S8; Supplementary Table S5). This helps explain the aggressive tendency of AVPC to progress and the relative inefficacy of NHT. Many of our patients had alterations in genes affecting *TP53*, *RB1*, or *PTEN* as well as alterations in DDR genes, consistent with Aparicio studies (43, 49, 50). The prevalence of DDR alterations in AVPC is controversial as tumor suppressor genes (*TP53*, *RB1*, and *PTEN*) and DDR genes were nearly mutually exclusive in treatment-emergent small-cell neuroendocrine prostate cancer (51), while it was reported that two more losses of *TP53*, *RB1*, and *PTEN* genes in CTCs were associated with increased genomic instability (43). Because the observed alterations in AVPC weaken the efficacy of AR-targeted therapy, the role of PARP inhibitors is currently being explored in clinical trials (NCT03263650 and NCT04592237). We also found a relatively high frequency of *CDK12* alterations in our patients, implying that they may respond to immunotherapy (28, 52). At the same time, few of our patients showed *PTEN* loss, implying that few of them would respond to dysregulated activity of the PI3K/AKT signaling cascade (53).

Similar to previous studies, we found quite short median time from initiation of ADT to CRPC in our patients with AVPC, indicating the androgen-independent biology of the cancer and the corresponding inefficacy of standard hormonal therapy (54). The most frequent cause of resistance to NHT may be alterations in *TP53*, *RB1*, and *PTEN* (28, 50, 55, 56). Alterations in *TP53* and *RB1* may lead to neuroendocrine differentiation (18, 57), while alterations in *PTEN* may activate AR signaling. Loss-of-function alterations in *TP53*, *RB1*, or *PTEN* usually promote unrestricted proliferation and cell cycling despite the presence of DNA damage (58). Platinum-based chemotherapy can arrest cell-cycle progression and induce apoptosis, and it has been shown to improve survival of patients with AVPC, especially those with alterations in *TP53*, *RB1*, or *PTEN* (6). Indeed, loss-of-function alterations in *TP53* or *RB1* have been associated with rapid resistance to docetaxel (28, 59), so we explored here whether combining platinum-based chemotherapy with docetaxel or administering platinum-based chemotherapy after progression on docetaxel could improve survival. We found that indeed, these approaches led to better PFS and OS benefits than docetaxel only. To have a reliable and effective conclusion, multiple test was expected to be employed for managing the overall type I error rate (39, 60). We control the FDR by using Benjamini and Hochberg method, and demonstrate robust performance in a variety of therapy settings. Furthermore, we confirm chemotherapy has a higher survival rate than NHT, and the benefit of additional platinum-based chemotherapy may depend on alterations in *TP53*, *RB1*, or *PTEN* and ctDNA proportion in patients with AVPC. Nevertheless, the survival benefit was still short-lived, highlighting the need to continue searching for more effective treatments, such as inhibitors of immune checkpoints (e.g., clinical trials NCT04709276 and NCT04388852) as well as inhibitors of PARP and ATR (18, 61).

Our findings still should be interpreted carefully in light of the small sample, single institution and their heterogeneous therapies, which prevented us from performing subgroup analyses based on histopathology and types of therapies. We sampled ctDNA only once, and our NGS detected only exons, so our analysis may have the possibility of failing to detect more clinically relevant alterations. Our results should be verified and extended in well-designed, large prospective trials, especially our analysis of the efficacy of platinum-based chemotherapy.

In conclusion, our study first provides real-world evidence for the need to combine the sequencing of ctDNA and tumor tissue, rather than relying solely on either of them when characterizing AVPC. Chemotherapy is associated with significantly better survival than NHT, and the benefit of additional platinum-based chemotherapy may depend on the presence of alterations in *TP53*, *RB1* or *PTEN* and on a sufficiently high proportion of ctDNA in patients with AVPC. Our study supports the potential of combining genomic alterations from tumor tissue and ctDNA for selecting therapeutic regimens and predicting the prognosis for patients with AVPC. Future work should verify our findings with a prospective design, and multi-omics and artificial intelligence might be helpful to identify prognostic biomarkers and therapeutic targets against AVPC.

## Supplementary Material

Supplementary Table 1 Source, Multi-modal Imaging Characterization and Sampling of Progressive Tumor Tissue for Next-generation Sequencing.

Supplementary Table 2 Somatic Mutations and Deleterious Germline Mutations Detected in Progressive Tumor Tissue and Matched Circulating Tumor DNA for Each AVPC Patient.

Supplementary Table 3 Copy Number Variants Detected in Progressive Tumor Tissue and Matched Circulating Tumor DNA for Each AVPC Patient.

Supplementary Table 4 Clinical Characteristics of AVPC Patients, Stratified by Systemic Therapy with or without Local Therapy

Supplementary Table 5 Overview of Published and Ongoing Clinical Studies of Patients with AVPC.

Supplementary Figure 1 Supplementary Figure 1. Characteristics of the study cohort at the patient level. (A) The presence of diagnostic criteria for AVPC in each patient. Criteria have been described in Materials and Methods. (B) Deleterious genomic alterations. (C) Treatment method. (D) Changes in PSA after treatment. (E) PFS and OS after treatment. #, truncated; *, level of PSA could not be evaluated.

Supplementary Figure 2 An additional calculation by using the selected patients (patients who have alteration detections in both tumor tissue and ctDNA in terms of the same alteration type) was developed for concordance.

Supplementary Figure 3 Assessing ctDNA% Predictive Ability for High Positive Concordance. Receiver operating characteristic curve to assess the ability of ctDNA% to predict high positive concordance when ctDNA% was treated as (A) a continuous variable or (B) binary variable, with the cut-off (13.5%) defined by the Youden index.

Supplementary Figure 4 Patient-level concordance of genomic alterations involving the tumor suppressor genes TP53, RB1 or PTEN between progressive tumor tissue and matched ctDNA in patients with AVPC.

Supplementary Figure 5 Ability of alterations affecting signaling pathways in progressive tumor tissue or matched ctDNA to predict PFS of patients with AVPC.

Supplementary Figure 6 Ability of alterations affecting signaling pathways in progressive tumor tissue or matched ctDNA to predict OS of patients with AVPC.

Supplementary Figure 7 Comparing Survival Outcomes in AVPC Subgroups: Additional Platinum-Based Chemo vs. Docetaxel-Only. Survival outcomes of PFS (A) and OS (B) for additional Platinum-based chemotherapy or Docetaxel only in the AVPC subgroups without any alteration involving the tumor suppressor genes TP53, RB1, or PTEN. Survival outcomes of PFS (C) and OS (D) for additional Platinum-based chemotherapy or Docetaxel only in the AVPC subgroups with low concentration of ctDNA (ctDNA% < 13.5%).

Supplementary Figure 8 Comparison between STPH cohort of patients with AVPC and patients from Aparicio et al and Corn et al studies. (A) Clinical characteristics. (B) Genomic alterations involving the tumor suppressor genes TP53, RB1 or PTEN.
