# Supplementary material for: Concordance and Clinical Significance of Genomic Alterations in Progressive Tumor Tissue and Matched Circulating Tumor DNA in Aggressive-variant Prostate Cancer
Source: Cancer Res Commun. 2023 Nov 3;3(11):2221–32. doi: 10.1158/2767-9764.CRC-23-0175 (PMC10624154; doi:10.1158/2767-9764.CRC-23-0175)
Supplement: Supplementary Figure 1 — Characteristics of the study cohort at the patient level. (A) The presence of diagnostic criteria for AVPC in each patient. Criteria have been described in Materials and Methods. (B) Deleterious genomic alterations. (C) Treatment method. (D) Changes in PSA after treatment. (E) PFS and OS after treatment. #, truncated; *, level of PSA could not be evaluated. [file crc-23-0175-s06.pdf]

Supplementary Figure 1

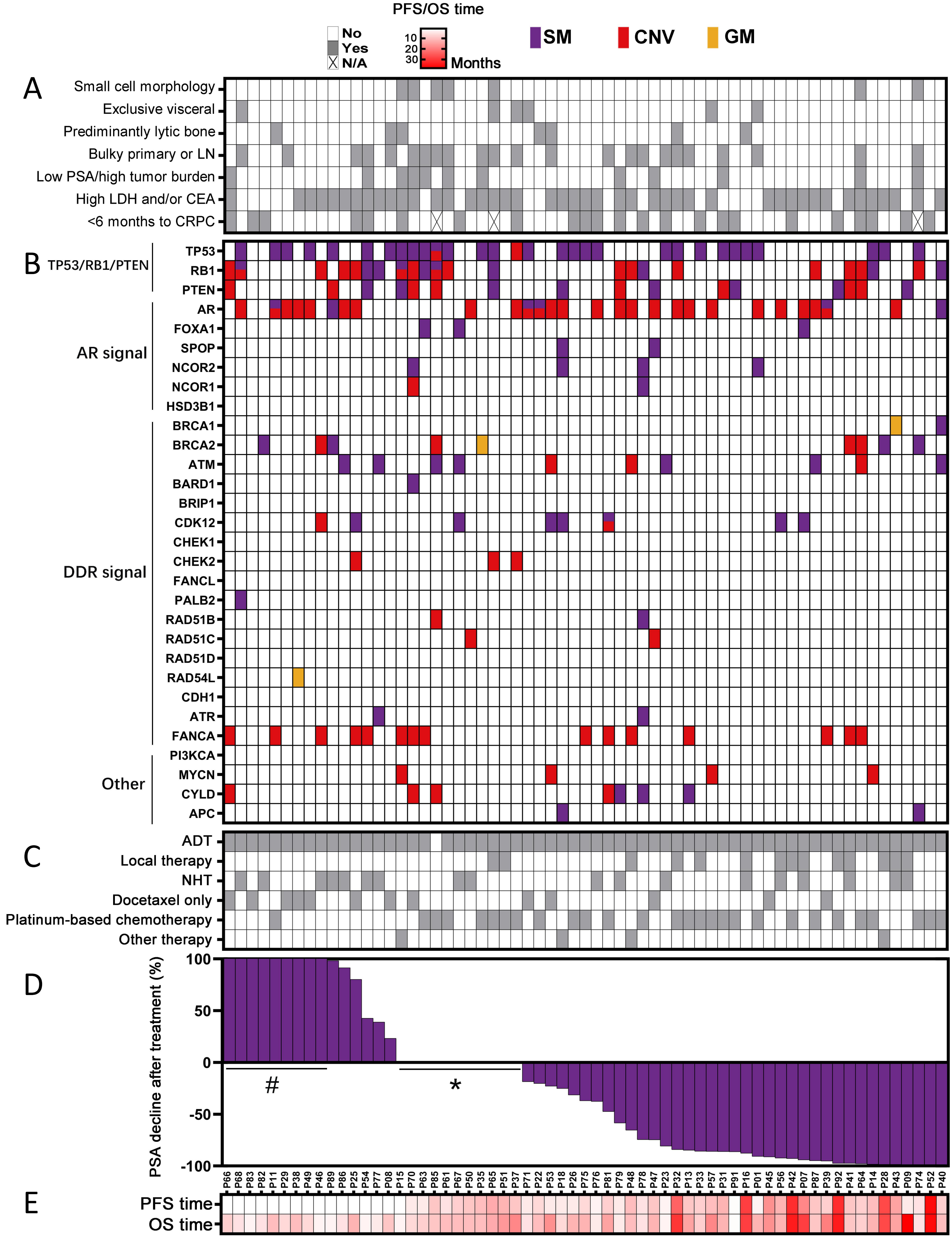

Supplementary Figure 1. Characteristics of the study cohort at the patient level. (A) The presence of diagnostic criteria for AVPC in each patient. Criteria have been described in Materials and Methods. (B) Deleterious genomic alterations. (C) Treatment method. (D) Changes in PSA after treatment. (E) PFS and OS after treatment. #, truncated; *, level of PSA could not be evaluated.
