# Supplementary material for: Concordance and Clinical Significance of Genomic Alterations in Progressive Tumor Tissue and Matched Circulating Tumor DNA in Aggressive-variant Prostate Cancer
Source: Cancer Res Commun. 2023 Nov 3;3(11):2221–32. doi: 10.1158/2767-9764.CRC-23-0175 (PMC10624154; doi:10.1158/2767-9764.CRC-23-0175)
Supplement: Supplementary Figure 2 — An additional calculation by using the selected patients (patients who have alteration detections in both tumor tissue and ctDNA in terms of the same alteration type) was developed for concordance. [file crc-23-0175-s07.pdf]

Supplementary Figure 2

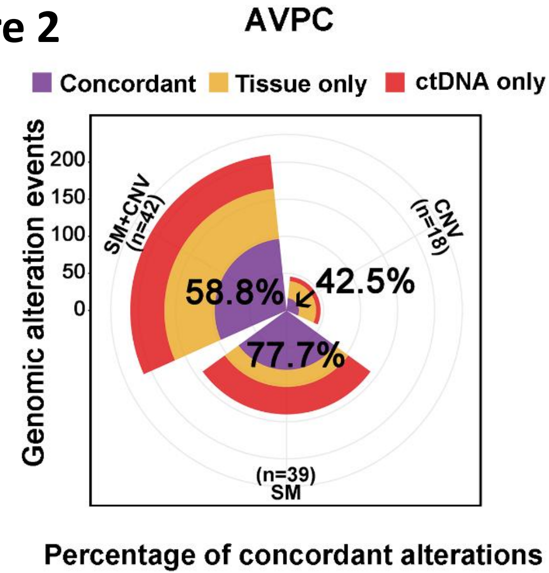

Supplementary Figure 2. An additional calculation by using the selected patients (patients who have alteration detections in both tumor tissue and ctDNA in terms of the same alteration type) was developed for concordance.
