# Supplementary material for: Concordance and Clinical Significance of Genomic Alterations in Progressive Tumor Tissue and Matched Circulating Tumor DNA in Aggressive-variant Prostate Cancer
Source: Cancer Res Commun. 2023 Nov 3;3(11):2221–32. doi: 10.1158/2767-9764.CRC-23-0175 (PMC10624154; doi:10.1158/2767-9764.CRC-23-0175)
Supplement: Supplementary Figure 3 — Assessing ctDNA% Predictive Ability for High Positive Concordance. Receiver operating characteristic curve to assess the ability of ctDNA% to predict high positive concordance when ctDNA% was treated as (A) a continuous variable or (B) binary variable, with the cut-off (13.5%) defined by the Youden index. [file crc-23-0175-s08.pdf]

Supplementary Figure 3

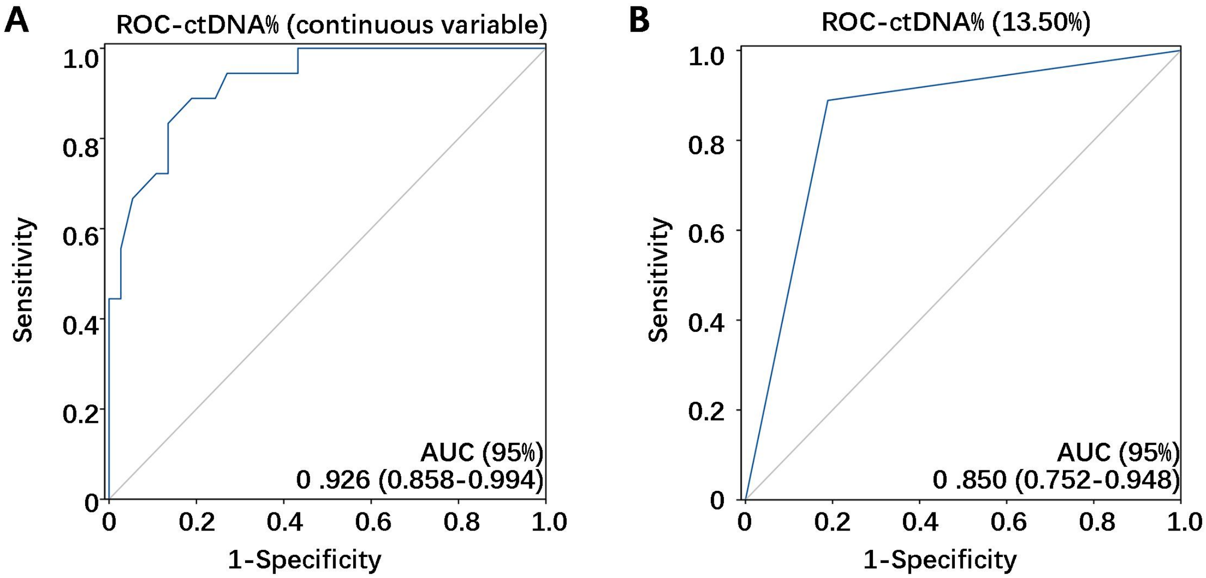

Supplementary Figure 3. Assessing ctDNA% Predictive Ability for High Positive Concordance. Receiver operating characteristic curve to assess the ability of ctDNA% to predict high positive concordance when ctDNA% was treated as (A) a continuous variable or (B) binary variable, with the cut-off (13.5%) defined by the Youden index.
