# Supplementary material for: Concordance and Clinical Significance of Genomic Alterations in Progressive Tumor Tissue and Matched Circulating Tumor DNA in Aggressive-variant Prostate Cancer
Source: Cancer Res Commun. 2023 Nov 3;3(11):2221–32. doi: 10.1158/2767-9764.CRC-23-0175 (PMC10624154; doi:10.1158/2767-9764.CRC-23-0175)
Supplement: Supplementary Figure 4 — Patient-level concordance of genomic alterations involving the tumor suppressor genes TP53, RB1 or PTEN between progressive tumor tissue and matched ctDNA in patients with AVPC. [file crc-23-0175-s09.pdf]

Supplementary Figure 4

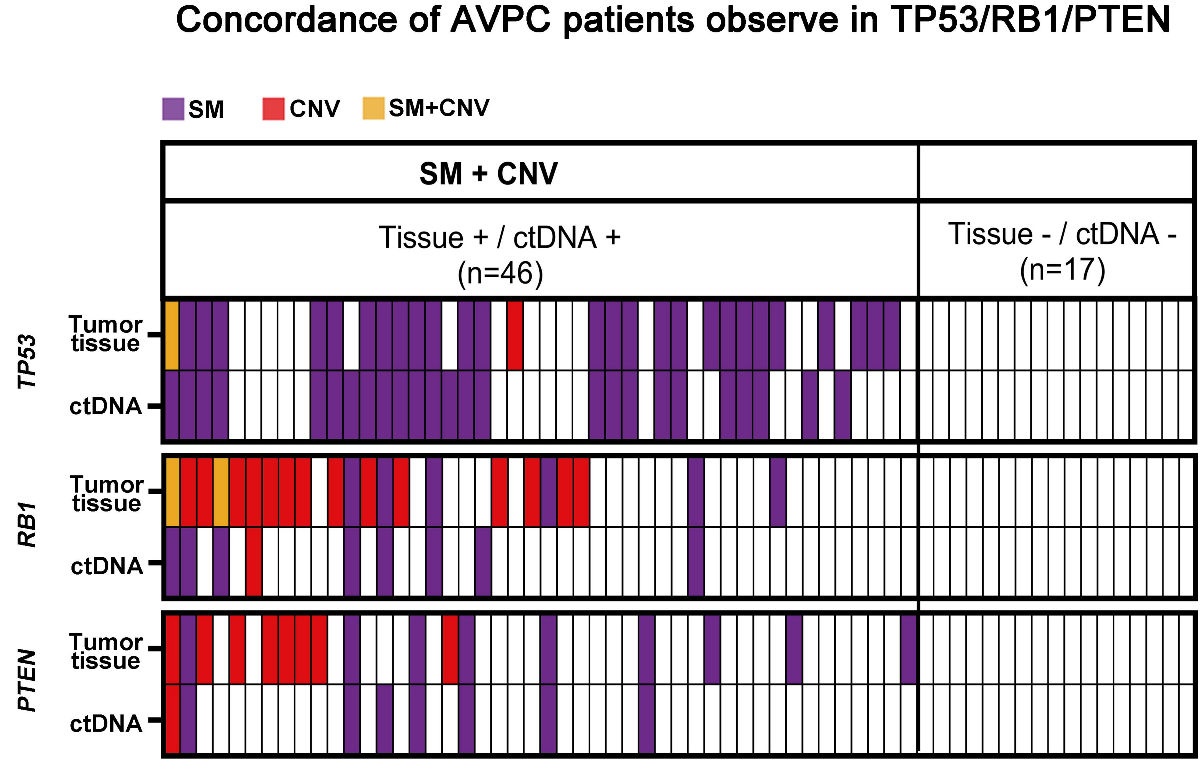

Supplementary Figure 4. Patient-level concordance of genomic alterations involving the tumor suppressor genes TP53, RB1 or PTEN between progressive tumor tissue and matched ctDNA in patients with AVPC.
