# Supplementary material for: Concordance and Clinical Significance of Genomic Alterations in Progressive Tumor Tissue and Matched Circulating Tumor DNA in Aggressive-variant Prostate Cancer
Source: Cancer Res Commun. 2023 Nov 3;3(11):2221–32. doi: 10.1158/2767-9764.CRC-23-0175 (PMC10624154; doi:10.1158/2767-9764.CRC-23-0175)
Supplement: Supplementary Figure 5 — Ability of alterations affecting signaling pathways in progressive tumor tissue or matched ctDNA to predict PFS of patients with AVPC. [file crc-23-0175-s10.pdf]

Supplementary Figure 5

Characteristics	Total (N)	HR (95% CI) Univariate analysis for OS	P value
**TP53/RB1/PTEN - Tissue**			0.038
No alteration	14	Reference	
Any alteration	35	2.752 (1.057-7.163)	
**AR Signal - Tissue**			0.432
No alteration	23	Reference	
Any alteration	26	1.397 (0.606-3.221)	
**DDR Siganal - Tissue**			0.625
No alteration	22	Reference	
Any alteration	27	1.225 (0.543-2.763)	
**Other genes - Tissue**			0.825
No alteration	42	Reference	
Any alteration	7	0.884 (0.298-2.626)	
**TP53/RB1/PTEN - ctDNA**			0.011
No alteration	28	Reference	
Any alteration	21	3.444 (1.327-8.938)	
**AR Signal - ctDNA**			0.633
No alteration	30	Reference	
Any alteration	19	1.243 (0.509-3.037)	
**DDR Signal - ctDNA**			0.041
No alteration	31	Reference	
Any alteration	18	2.476 (1.038-5.903)	
**Other genes - ctDNA**			0.557
No alteration	41	Reference	
Any alteration	8	1.350 (0.496-3.679)	
**TP53/RB1/PTEN - Aggregation**			0.027
No alteration	13	Reference	
Any alteration	36	3.134 (1.140-8.615)	
**AR Signal - Aggregation**			0.700
No alteration	22	Reference	
Any alteration	27	1.177 (0.513-2.704)	
**DDR Signal - Aggregation**			0.472
No alteration	17	Reference	
Any alteration	32	1.368 (0.582-3.214)	
**Other genes - Aggregation**			0.855
No alteration	36	Reference	
Any alteration	13	1.088 (0.443-2.672)	

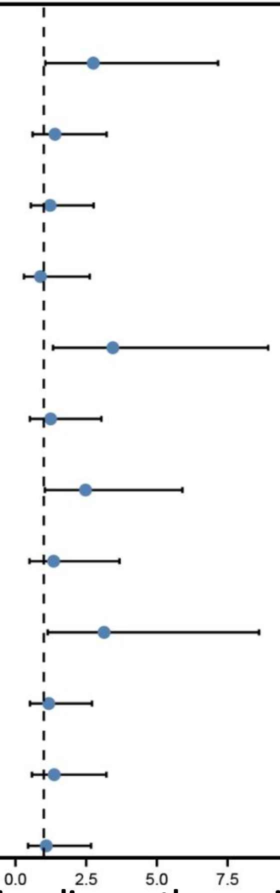

Supplementary Figure 5. Ability of alterations affecting signaling pathways in progressive tumor tissue or matched ctDNA to predict PFS of patients with AVPC.
