# Supplementary material for: Concordance and Clinical Significance of Genomic Alterations in Progressive Tumor Tissue and Matched Circulating Tumor DNA in Aggressive-variant Prostate Cancer
Source: Cancer Res Commun. 2023 Nov 3;3(11):2221–32. doi: 10.1158/2767-9764.CRC-23-0175 (PMC10624154; doi:10.1158/2767-9764.CRC-23-0175)
Supplement: Supplementary Figure 6 — Ability of alterations affecting signaling pathways in progressive tumor tissue or matched ctDNA to predict OS of patients with AVPC. [file crc-23-0175-s11.pdf]

Supplementary Figure 6

Characteristics	Total (N)	HR (95% CI) Univariate analysis for PFS	P value
**TP53/RB1/PTEN - Tissue**			0.056
No alteration	14	Reference	
Any alteration	35	2.044 (0.982-4.257)	
**AR Signal - Tissue**			0.127
No alteration	23	Reference	
Any alteration	26	1.648 (0.868-3.130)	
**DDR Siganal - Tissue**			0.601
No alteration	22	Reference	
Any alteration	27	1.184 (0.629-2.228)	
**Other genes - Tissue**			0.615
No alteration	42	Reference	
Any alteration	7	0.799 (0.333-1.917)	
**TP53/RB1/PTEN - ctDNA**			0.352
No alteration	28	Reference	
Any alteration	21	1.355 (0.714-2.571)	
**AR Signal - ctDNA**			0.328
No alteration	30	Reference	
Any alteration	19	1.378 (0.725-2.619)	
**DDR Signal - ctDNA**			0.626
No alteration	31	Reference	
Any alteration	18	0.848 (0.437-1.646)	
**Other genes - ctDNA**			0.444
No alteration	41	Reference	
Any alteration	8	0.711 (0.296-1.704)	
**TP53/RB1/PTEN - Aggregation**			0.041
No alteration	13	Reference	
Any alteration	36	2.209 (1.032-4.729)	
**AR Signal - Aggregation**			0.143
No alteration	22	Reference	
Any alteration	27	1.626 (0.849-3.114)	
**DDR Signal - Aggregation**			0.594
No alteration	17	Reference	
Any alteration	32	1.198 (0.616-2.329)	
**Other genes - Aggregation**			0.260
No alteration	36	Reference	
Any alteration	13	0.660 (0.321-1.360)	

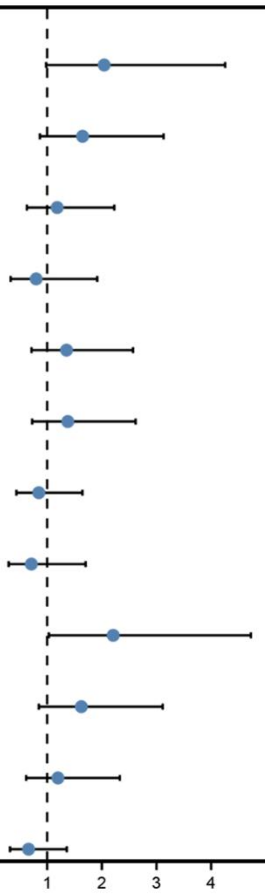

Supplementary Figure 6. Ability of alterations affecting signaling pathways in progressive tumor tissue or matched ctDNA to predict OS of patients with AVPC.
