# Supplementary material for: Concordance and Clinical Significance of Genomic Alterations in Progressive Tumor Tissue and Matched Circulating Tumor DNA in Aggressive-variant Prostate Cancer
Source: Cancer Res Commun. 2023 Nov 3;3(11):2221–32. doi: 10.1158/2767-9764.CRC-23-0175 (PMC10624154; doi:10.1158/2767-9764.CRC-23-0175)
Supplement: Supplementary Figure 7 — Comparing Survival Outcomes in AVPC Subgroups: Additional Platinum-Based Chemo vs. Docetaxel-Only. Survival outcomes of PFS (A) and OS (B) for additional Platinum-based chemotherapy or Docetaxel only in the AVPC subgroups without any alteration involving the tumor suppressor genes TP53, RB1, or PTEN. Survival outcomes of PFS (C) and OS (D) for additional Platinum-based chemotherapy or Docetaxel only in the AVPC subgroups with low concentration of ctDNA (ctDNA% < 13.5%). [file crc-23-0175-s12.pdf]

Supplementary Figure 7

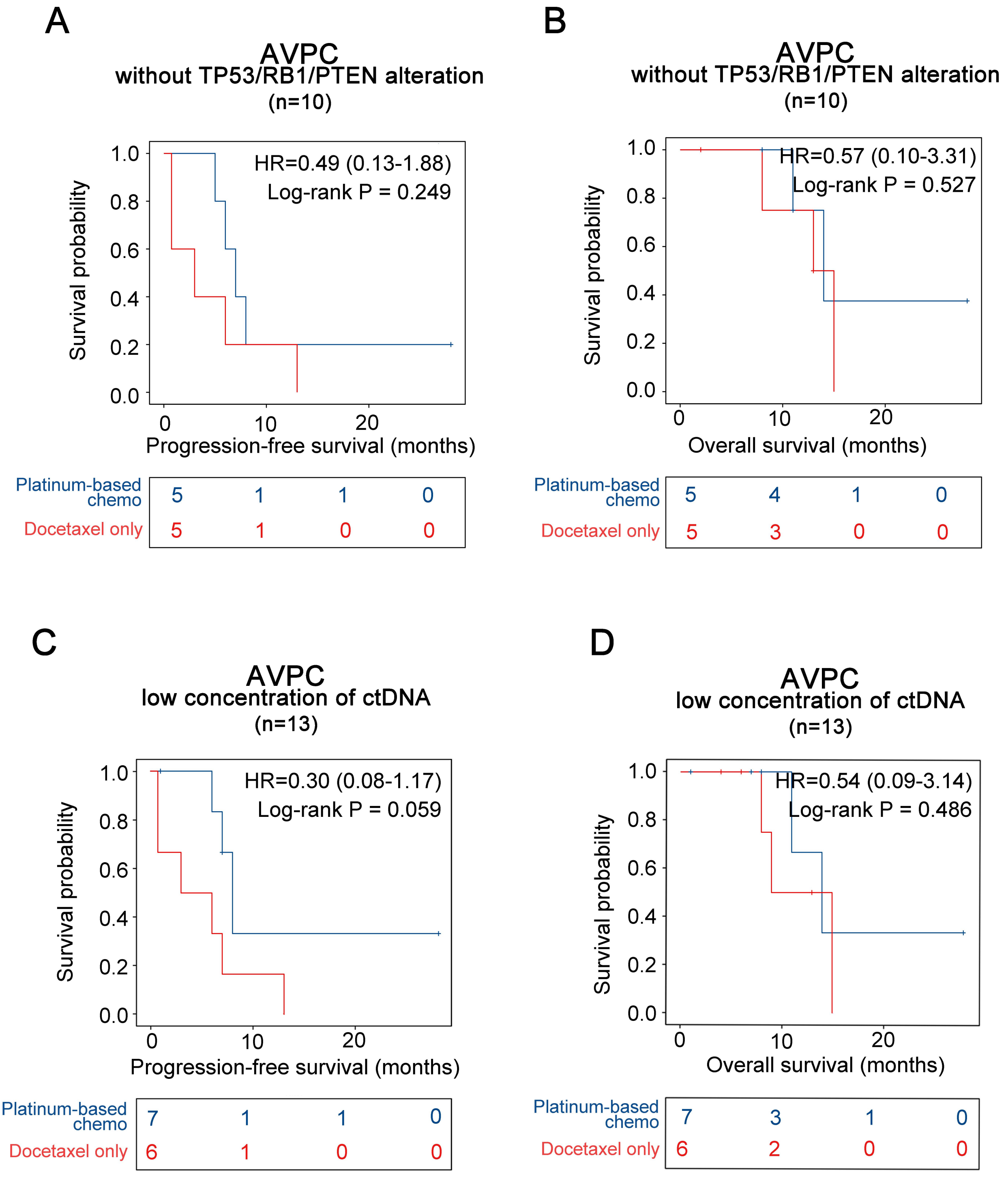

Supplementary Figure7: Comparing Survival Outcomes in AVPC Subgroups: Additional Platinum-Based Chemo vs. Docetaxel-Only. Survival outcomes of PFS (A) and OS (B) for additional Platinum-based chemotherapy or Docetaxel only in the AVPC subgroups without any alteration involving the tumor suppressor genes TP53, RB1, or PTEN. Survival outcomes of PFS (C) and OS (D) for additional Platinum-based chemotherapy or Docetaxel only in the AVPC subgroups with low concentration of ctDNA (ctDNA% < 13.5%).
