# Supplementary material for: Concordance and Clinical Significance of Genomic Alterations in Progressive Tumor Tissue and Matched Circulating Tumor DNA in Aggressive-variant Prostate Cancer
Source: Cancer Res Commun. 2023 Nov 3;3(11):2221–32. doi: 10.1158/2767-9764.CRC-23-0175 (PMC10624154; doi:10.1158/2767-9764.CRC-23-0175)
Supplement: Supplementary Figure 8 — Comparison between STPH cohort of patients with AVPC and patients from Aparicio et al and Corn et al studies. (A) Clinical characteristics. (B) Genomic alterations involving the tumor suppressor genes TP53, RB1 or PTEN. [file crc-23-0175-s13.pdf]

Supplementary Figure 8

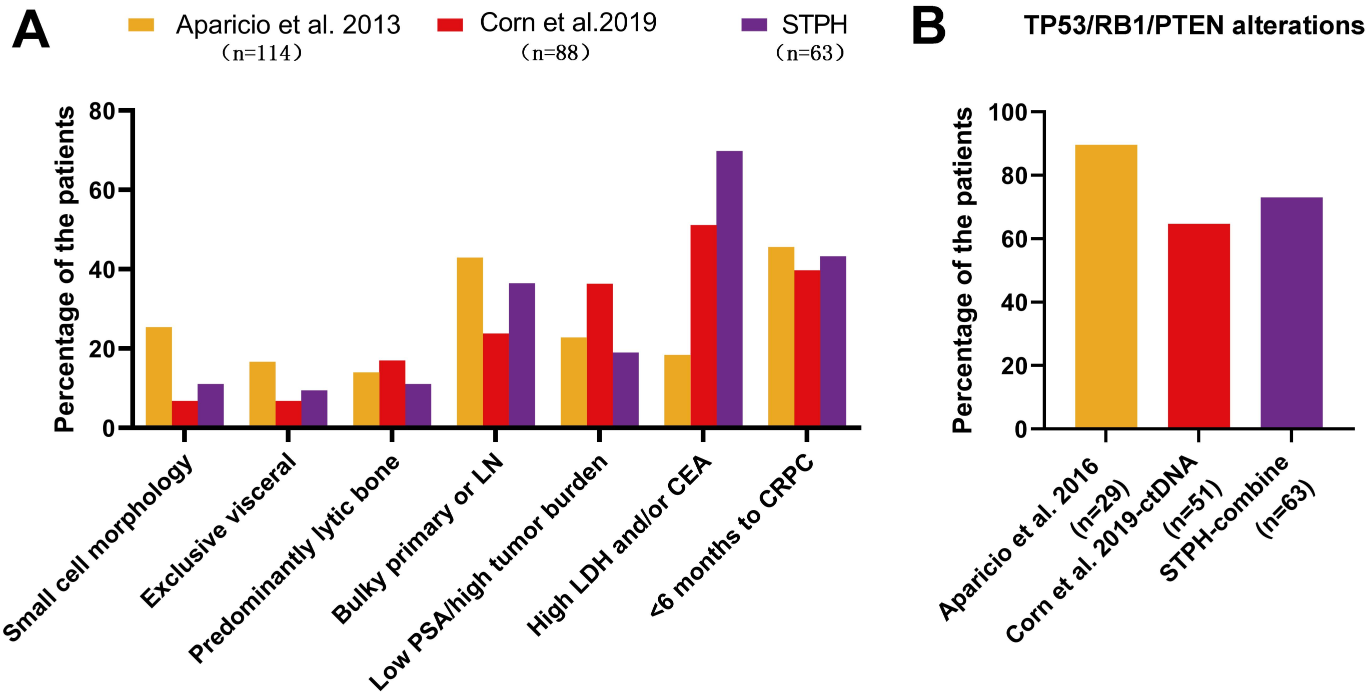

Supplementary Figure 8. Comparison between STPH cohort of patients with AVPC and patients from Aparicio et al and Corn et al studies. (A) Clinical characteristics. (B) Genomic alterations involving the tumor suppressor genes TP53, RB1 or PTEN.
